# An Atypical Presentation of Goldenhar Syndrome With Seizures: A Rare Case Report

**DOI:** 10.7759/cureus.46627

**Published:** 2023-10-07

**Authors:** Shivangi C Tidke, Jayant D Vagha, Keta Vagha, Sham Lohiya, Priyanka Hampe

**Affiliations:** 1 Pediatrics, Jawaharlal Nehru Medical College, Datta Meghe Institute of Higher Education and Research, Wardha, IND

**Keywords:** esophageal stricture, preauricular tags, seizures, microtia, oculo-auriculo-vertebral spectrum

## Abstract

The Goldenhar syndrome also known as oculo-auriculo-vertebral dysplasia is one of the rare congenital defects that usually involves the impaired development of structures derived from first and second branchial arches such as ears, eyes, mandible, palate and various other structures of the face along with spinal abnormalities. The severity of Goldenhar syndrome anomalies can range from minor to severe, and patients with modest facial asymmetry to those with a highly evident facial abnormality. The most typical characteristics of this condition are dental ailments and impaired development of the mandible, maxilla, zygomatic, orbital, lips, tongue, and palate. It may also include hemifacial microsomia along with the cleft lip or cleft palate. The aetiology may include genetic and environmental factors but in most of the cases, the aetiology remains unknown. Gestational diabetes mellitus is also one of the leading risk factors associated with Goldenhar syndrome. The treatment and management depend upon the age of the patient and the clinical presentation. This case report describes an eight-year-old male child with generalised tonic-clonic seizures in all the limbs along with peri auricular skin tags, mandibular hypoplasia and esophageal stricture. There were no ocular findings or vertebral deformities.

## Introduction

The Goldenhar syndrome also known as oculo-auriculo-vertebral spectrum was first reported by Maurice Goldenhar in 1952 [1]. Goldenhar syndrome can also be called as first and second arch syndrome as the structures involved are derived from the branchial arches [1,2]. The pathogenesis of the disease is mediated by MSX homeobox genes but its exact etiology still remains unknown. Most of the defects are unilateral among which the right side is affected in most patients [3]. The spectrum of Goldenhar syndrome ranges from mild to severe including patients with barely noticeable facial asymmetry to very pronounced and severe facial defects with skeletal abnormalities. It is characterized most commonly by impaired development of eyes, ears, lips, tongue, palate, mandible, maxilla, zygomatic and orbital structures and deformations of the dental structures [1,4]. It may also involve cardiac syndromes, central nervous system involvement, trachea and lung malformations, and kidney and gastrointestinal defects. The incidence was observed to be between 1:3500 and 1:5600 and males are affected more commonly than females with a ratio of 3:2 [4]. The incidence of the disease is also associated with the consumption of retinoic acid and primidone in the antenatal period.

## Case presentation

An eight-year-old male child presented to the Department of Pediatrics with generalized tonic-clonic seizure (GTCS) type of convulsions involving all four limbs with up rolling of the eyes that lasted for about 10 minutes. He has been unable to feed properly for the last two months due to pain while swallowing. He was born of third-degree consanguineous marriage and was diagnosed with right microtia, left-sided anotia and facial asymmetry at birth along with Tetralogy of Fallot which was managed medically for three years. The patient was also found to have developmental delays.

**Figure 1:**
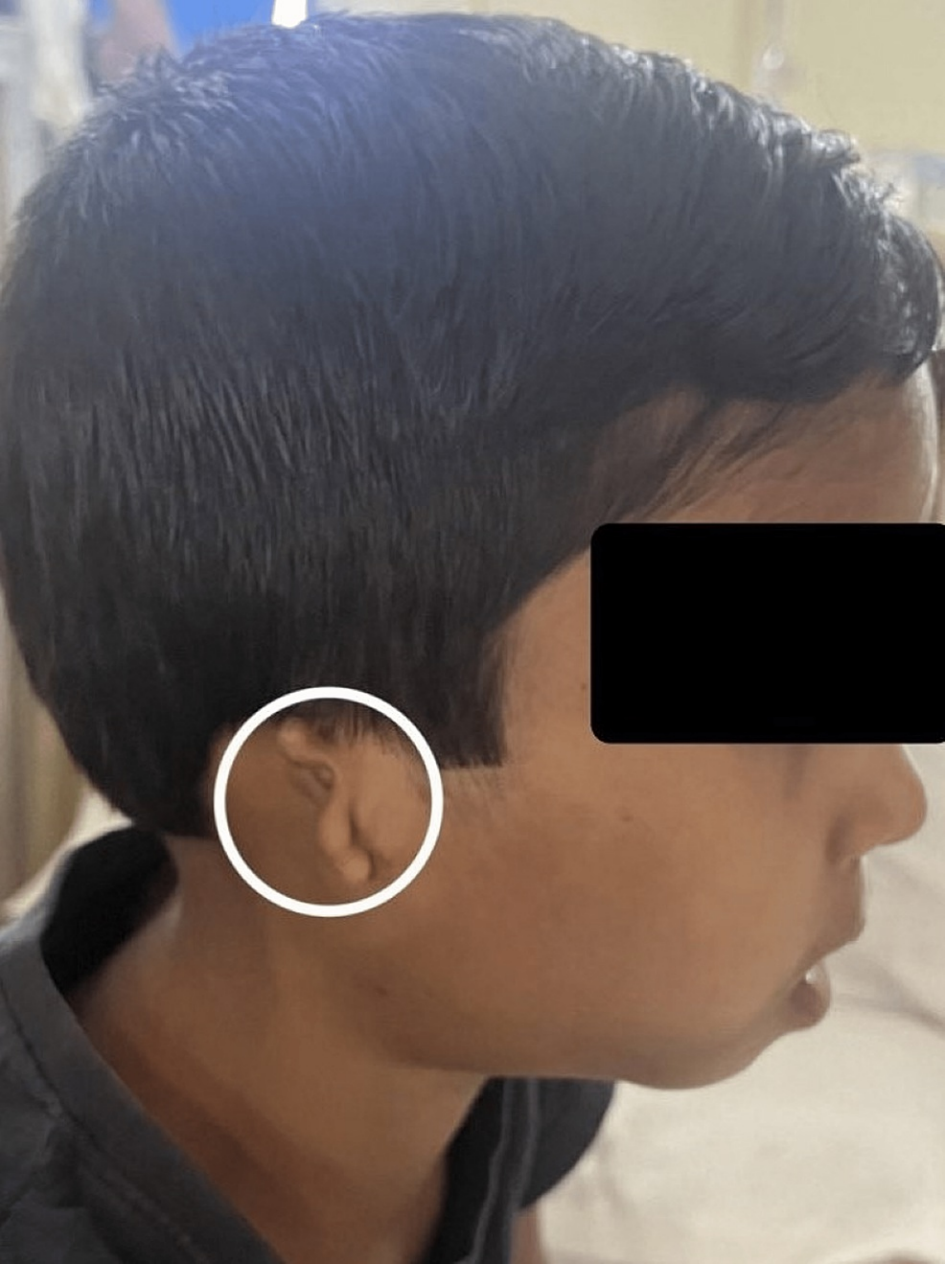
Microtia of the right ear

**Figure 2:**
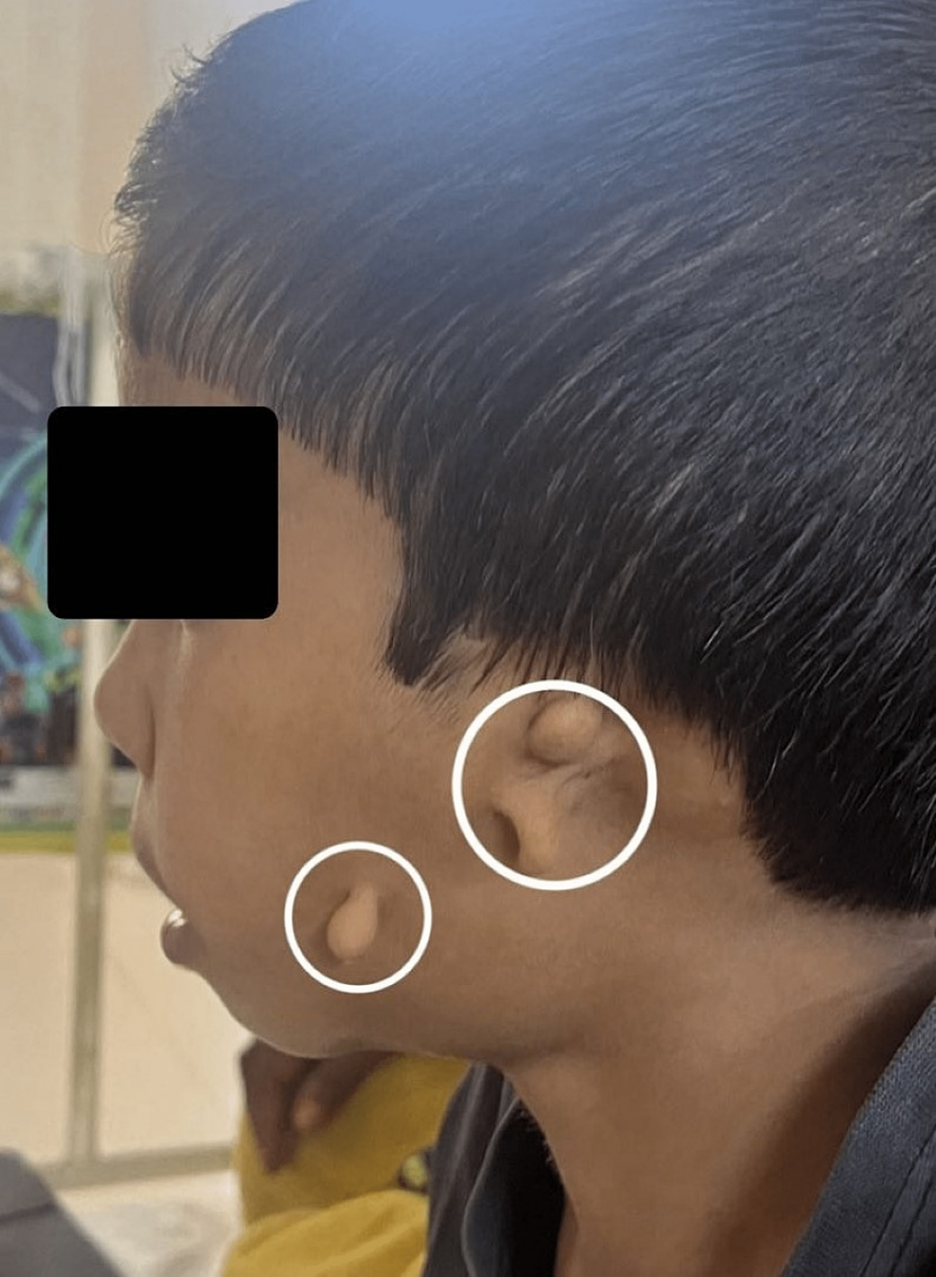
Lateral view showing peri auricular skin tags on left side

**Figure 3:**
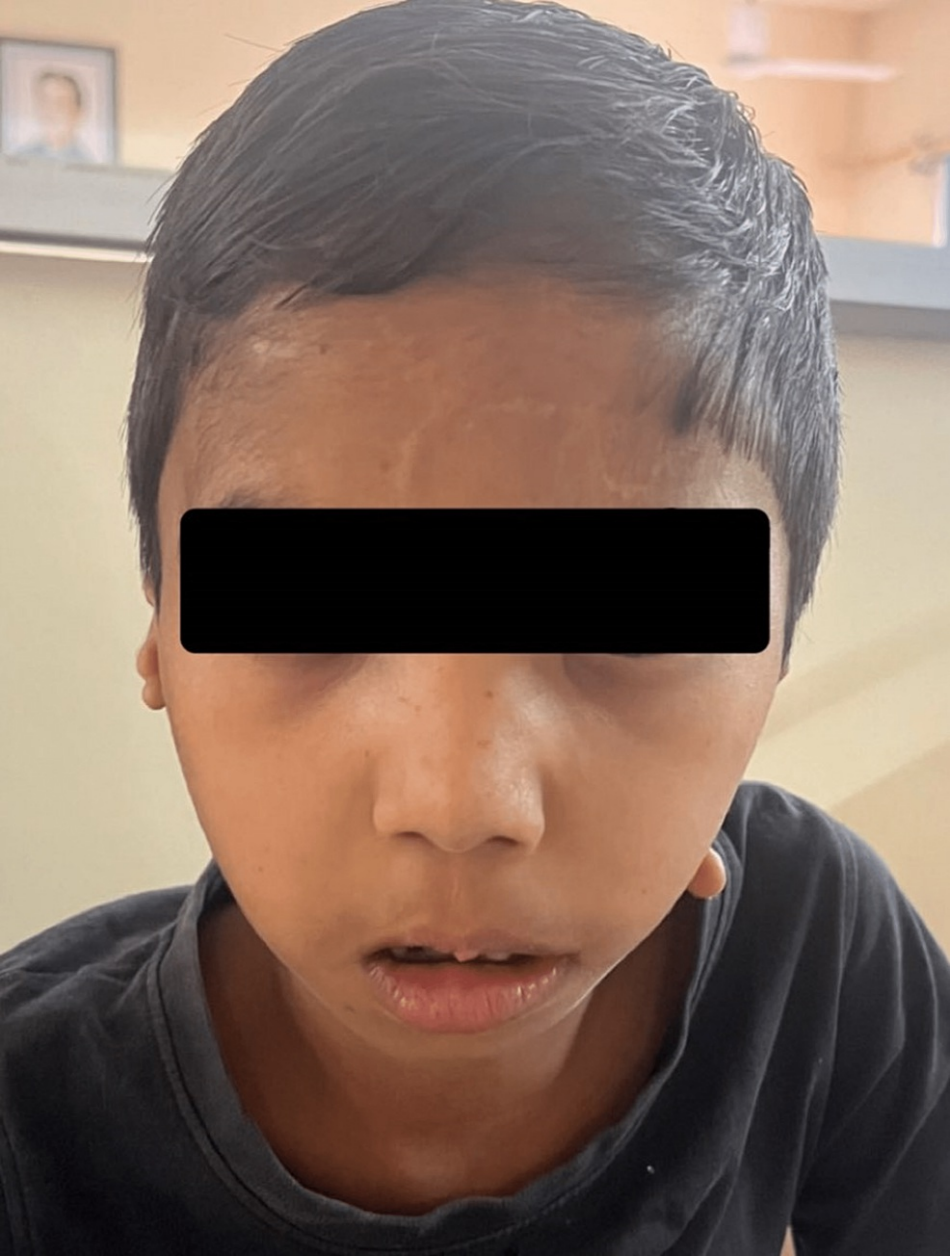
Frontal view showing deviation of the mouth to right side along with mandibular hypoplasia

Figure 1 shows skin tags with absent ear cartilage over the left side of the face. Figure 2 shows microtia with swelling over the right ear. The external auditory meatus opening was absent. Mandibular hypoplasia was seen on both sides of the face as shown in Figure 3. The audiogram shows bilateral conductive hearing loss. On central nervous system (CNS) examination, partial closure of the left eye along with the deviation of the mouth to the right side was seen due to facial nerve palsy. On cardiovascular examination, the heart sounds were auscultated with no murmurs. The ocular examination showed no abnormal findings. There were no limbal dermoids.

**Figure 4:**
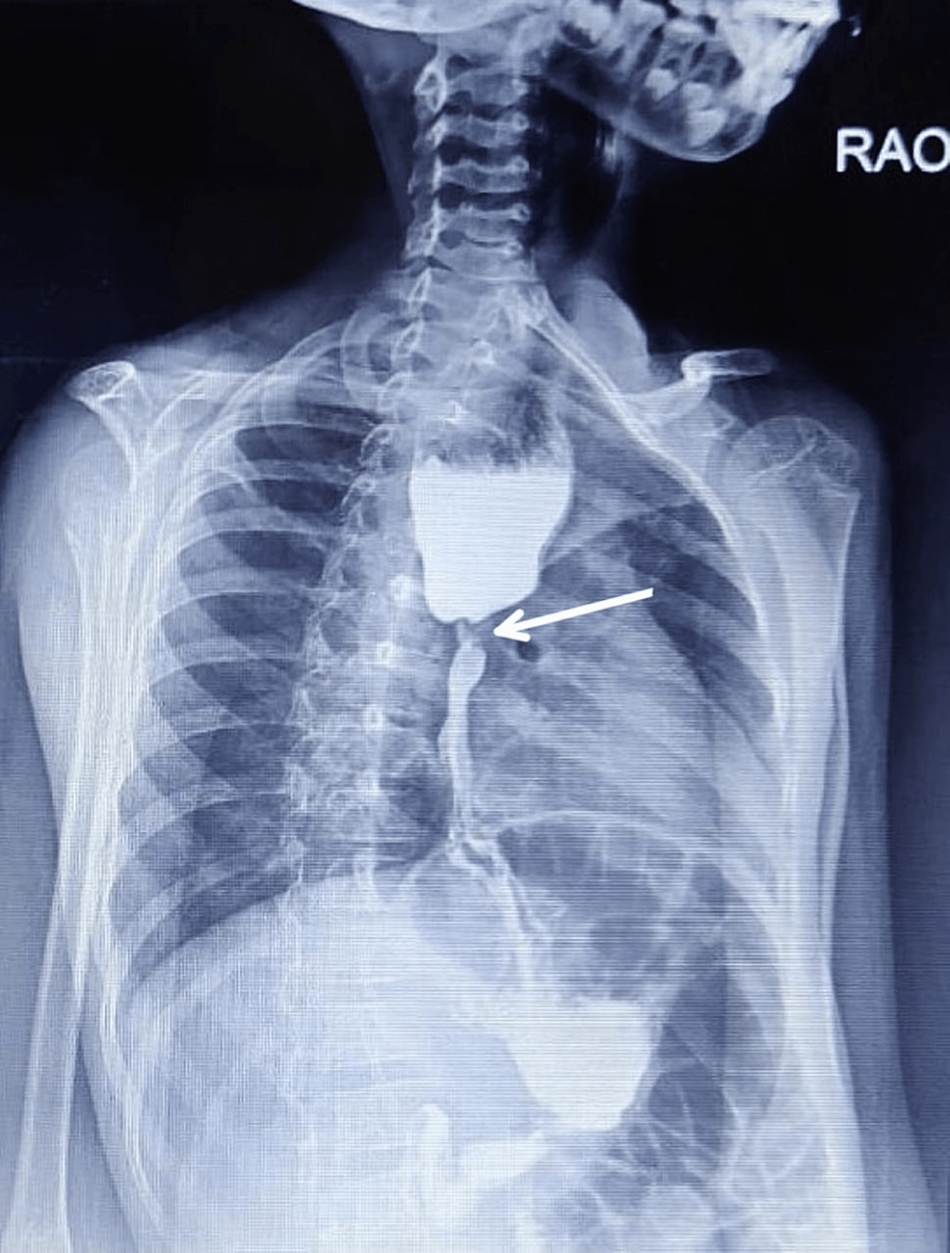
Radiological findings of Goldenhar syndrome on barium swallow showing esophageal stricture

Endoscopy shows tight stricture of the esophagus approximately 2 cm noted at 20 cm from the incisor tooth. The child was followed up with esophageal dilatation. The graded dilatation was performed from 5 mm up to 11 mm. Further dilatation could not be done due to respiratory distress. The CT scan external auditory canal is not visualized bilaterally. The bilateral pinna appears tiny and deformed. The mandibular condyle appears smaller on the left as compared to the right. There is found to be asymmetrical pneumatization of mastoid air cells with the left side more pneumatized as compared to the right. The barium swallow showing a massively enlarged esophagus followed by esophageal stricture as shown in Figure 4.

## Discussion

Goldenhar syndrome is a condition that can cause a variety of physical and developmental symptoms in children. The common presentation includes ocular and auricular abnormalities along with differences in facial bone structure. Children with Goldenhar syndrome might also experience delayed development, speech and psycho-social problems, and occasionally display autistic behaviours. Other symptoms can include low height and microphthalmia [5,6]. In this case, the child was additionally diagnosed with esophageal stricture leading to difficulty in feeding. It was managed with an esophageal dilatation procedure. He also had episodes of seizures which is one of the rare findings in the clinical presentation of Goldenhar syndrome.

The precise mechanisms underlying the development of Goldenhar syndrome remain poorly understood, with many cases still being unexplained. The most probable cause of Goldenhar syndrome is thought to be the infectious features like antibodies against cytomegalovirus, rubella or herpes simplex virus [7]. Sometimes lacrimal canal anomalies can be also seen due to obstruction of the canal or the obstruction of the nasolacrimal duct [8]. Nonetheless, advances in genetic research have shed light on this congenital condition, revealing it to be a complex interplay of environmental and genetic factors [1]. Familial cases with autosomal dominant or recessive inheritance have been documented in the literature, indicating a likely multifactorial cause [9]. It is found that autosomal dominant genetic inheritance is more common as compared to less frequent autosomal recessive transmission [8]. The mainstay for the diagnosis of the disease is based upon the clinical presentation, laboratory and radiological findings of the patient. In some cases, the external eye is involved with rare involvement of the retina leading to retinal detachment [10]. In this case, the patient is found with auricular, ocular and cardiac abnormalities along the facial nerve palsy showing deviation of mouth and partial closure of the eye.

## Conclusions

Goldenhar syndrome is a rare congenital disorder involving various environmental and genetic risk factors with the involvement of multiple organs. The auricular involvement leads to delayed speech development. As a result, a precise diagnosis along with extensive multidisciplinary care is required to provide the most significant results for the patient. To ensure the best possible development for children with Goldenhar syndrome, effective collaboration between other doctors in the fields of ENT, medical rehabilitation, and pediatric neurology is crucial.
